# Acetyl Zingerone: A Photostable Multifunctional Skincare Ingredient That Combats Features of Intrinsic and Extrinsic Skin Aging

**DOI:** 10.3390/antiox12061168

**Published:** 2023-05-29

**Authors:** Thomas A. Meyer, William R. Swindell, Ratan K. Chaudhuri

**Affiliations:** 1 Sytheon, 10 Waterview Blvd, Parsippany, NJ 07054, USA; 2 Department of Internal Medicine, University of Texas Southwestern Medical Center, Dallas, TX 75390, USA; william.swindell@utsouthwestern.edu

**Keywords:** acetyl zingerone, non-sacrificial antioxidant, anti-inflammatory, selective chelator, incident and delayed CPDs, matrisome modulator, anti-senescence, tetrahexyldecyl ascorbate, clinical studies, skin microbiome

## Abstract

The cumulative damage skin sustains from exposure to environmental stressors throughout life exerts significant effects on skin aging and cancer development. One of the main ways by which environmental stressors mediate their effects within skin is through induction of reactive oxygen species (ROS). In this review, we chronicle the multiple properties by which acetyl zingerone (AZ) as a skincare ingredient can benefit skin (1) by helping manage overproduction of ROS through multiple routes as an antioxidant, physical quencher and selective chelator, (2) by fortifying protection after UV exposure ends to prevent the type of epidermal DNA damage that correlates with development of skin cancer, (3) by modulating matrisome activity and nurturing the integrity of the extracellular matrix (ECM) within the dermis and (4) through its proficient ability to neutralize singlet oxygen, by stabilizing the ascorbic acid precursor tetrahexyldecyl ascorbate (THDC) in the dermal microenvironment. This activity improves THDC bioavailability and may blunt pro-inflammatory effects of THDC, such as activation of type I interferon signaling. Moreover, AZ is photostable and can sustain its properties during UV exposure, in contrast to α-tocopherol. All these properties of AZ translate into measurable clinical benefits to improve the visual appearance of photoaged facial skin and to strengthen the skin’s own defenses against sun damage.

## 1. Introduction

With its intimate connection to the outside world, skin needs to defend itself constantly against a highly oxidizing environment [1]. The most relevant environmental stressors known to induce skin damage include solar radiation and air pollution (particulate matter and ozone), in addition to cigarette smoke, climate and lifestyle factors (diet and sleeping patterns) [2,3,4]. The cumulative damage that skin sustains over the course of life from exposure to these environmental stressors exerts significant effects on skin aging and development of skin cancer [5,6,7]. Moreover, recent studies have suggested a potential link between breached barrier function and progression of systemic chronic inflammatory disorders (including atherosclerosis, type II diabetes, osteoporosis, and Alzheimer’s disease) that may accompany advancement into older age. Together, these results suggest that maintaining skin in optimal condition may not only affect skin’s own health and appearance, but also impact overall health as humans progress into older age [8].

Prior work has extensively documented [1,2,3,4,5,6,7,9,10,11,12,13] that one of the main ways by which environmental stressors mediate their effects in skin is through induction of reactive active oxygen species (ROS). ROS constitutes a collection of highly reactive chemical entities, which, if left unchecked, can oxidize key cellular targets (lipids, proteins, DNA, and mitochondria), affect cellular signaling, induce inflammation and influence gene expression pathways. While skin possesses an elaborate network of enzymatic and non-enzymatic antioxidant defenses, studies show that these defenses are inherently weak and easily overwhelmed by the sheer abundance of ROS generated within skin upon exposure to environmental stressors, especially solar radiation, and ambient pollution [12,14,15]. When skin’s antioxidant defenses become depleted, the resulting excess of ROS leads to oxidative stress, a condition that perturbs the skin’s barrier function (loss of hydration, increased susceptibility to irritants and pathogens), accelerates premature skin aging (fine lines, wrinkles, loss of elasticity, uneven pigmentation, telangiectasia), provokes various dermatological disorders (assorted photodermatoses, atopic dermatitis, psoriasis, vitiligo) and facilitates development of melanoma and non-melanoma skin cancers [7,16,17].

Much of the skin damage induced by exposure to environmental stressors, however, can be greatly mitigated or prevented by application of topical products that contain the right mix of specialized ingredients. These include moisturizing agents to help maintain skin’s hydration and barrier function [18], sunscreen active ingredients to protect against the harmful effects of sun’s ultraviolet (UV) radiation [19] and efficacious ingredients with capacity either to neutralize the deleterious effects of ROS before they can induce damage within skin (i.e., antioxidants) or to work upstream of ROS formation to prevent their formation in the first place (i.e., physical quenchers) [20,21].

Acetyl zingerone (AZ) represents a new kind of skincare active ingredient with the ability to function proactively in multiple ways to help optimize skin’s overall health and appearance. In this review, we chronicle AZ’s multidimensional properties as a skincare active ingredient that contributes to its ability to boost the types of skin defenses needed to thwart premature aging, stimulate visible improvements to the manifestations of photoaging, and to bolster extra protection against the type of DNA damage that correlates with skin cancer development.

## 2. AZ Possesses a Unique Molecular Structure

### 2.1. Origin of Acetyl Zingerone

The chemical synthesis of Acetyl zingerone (AZ) has been described [22]. Briefly, vanillin (4-hydroxy-3-methoxybenzaldehyde) can be condensed with acetylacetone to obtain 3-(4-hydroxy, 3-methoxybenzylidene)-pentane-2,4-dione as an intermediate that is then catalytically hydrogenated to obtain AZ. This process can yield AZ with purity that exceeds 99% by HPLC (typically ranging from 99.3 to 99.9%), with structure confirmed by ^1^HNMR, ^13^CNMR and mass spectral analysis. Acetyl zingerone (trade name: Synoxyl^®^ AZ) is commercially available from Sytheon, Parsippany, NJ, USA. AZ is patent-protected (US 10,568,819, US 10,828,241 and other pending US and International patents), REACH-registered (EC# 820-605-0) and is safe for topical applications (as documented by the studies available for review on ECHA website).

### 2.2. Molecular Features of AZ

**Figure 1:**
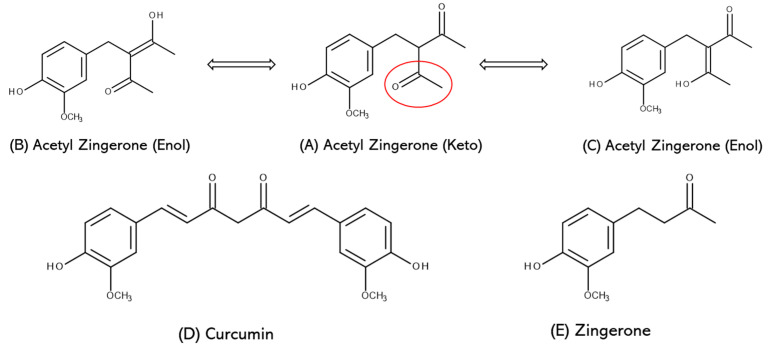
Acetyl zingerone exists as a mixture of (**A**) keto (62 ± 4%) and (**B**,**C**) enol (36 ± 4%) tautomers in methanol as determined by HPLC. The design of acetyl zingerone was inspired by the molecular structures of Curcumin (**D**) and Zingerone (**E**) (adapted from Chaudhuri et al. [22]).

AZ’s multifunctional properties originate from its unique chemical structure (3-[4-hydroxy-3-methoxybenzyl]pentane-2,4-dione) which was designed to bear structural resemblance to zingerone (4-[4-hydroxy-3-methoxyphenyl]-2-butanone) and curcumin (1,7-bis [4-hydroxy-3-methoxyphenyl]-1,6-heptane-3,5-dione), two highly efficacious natural skincare ingredients with well-documented antioxidant, anti-inflammatory and anti-aging properties [23,24,25,26,27,28,29,30]. Thus, AZ contains the 4-hydroxy-3-methoxyphenyl ring present in both zingerone and curcumin as well as the 1,3-di-carbonyl moiety present only in curcumin (Figure 1). While it retains many properties of zingerone and curcumin, AZ’s hybrid structure also exhibits improved photochemical stability, especially in comparison to curcumin, which is highly susceptible to degradation under solar radiation [31].

## 3. AZ Is Photochemically Stable and Can Deliver Sustained Benefits

**Figure 2:**
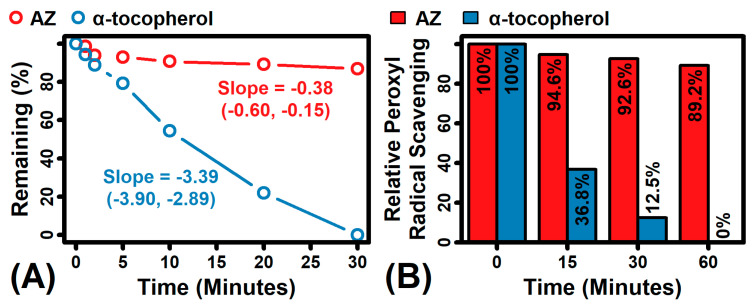
AZ versus α-tocopherol photostability comparison. (**A**) AZ is more chemically stable than α-tocopherol (adapted from Chaudhuri et al. [22]. The estimated decay slope is shown with 95% confidence intervals. (**B**) AZ sustains while α-tocopherol completely loses the ability to neutralize peroxyl radicals in a manner that mirrors their chemical stability when they are exposed to solar-simulated UV radiation over a time course of 1 h (1 h = 23 J/cm^2^).

Photochemical stability is an essential molecular property that skincare ingredients must possess to provide sustained benefits, particularly since such products are often be applied to sun-exposed body regions. When molecules are not stable photochemically, they undergo alterations to their chemical structures after they absorb the sun’s radiation that can disrupt their ability to provide sustained benefits. This is illustrated by the photostability profiles in Figure 2, which show that (1) the chemical integrity of AZ remains relatively intact compared with α-tocopherol (Figure 2A), which completely degrades over an irradiation time course [22] and (2) the relative ability to sustain neutralization of peroxyl radicals during irradiation (Figure 2B) is maintained by AZ but not by α-tocopherol in a manner that mirrors changes to their own chemical structures during irradiation (unpublished data). These results support the proposed benefits of AZ as an effective ingredient for use in topical products such as sunscreens as well as daily skincare products, applied to areas of skin that routinely receive sun exposure.

## 4. AZ Manages Excess ROS Formation through Multiple Routes

The overproduction of ROS in skin stimulated by exposure to environmental stressors can be managed by topical application of specialized ingredients. This has mostly been achieved with antioxidants that function to neutralize ROS once formed but before they can inflict damage [32]. To be effective, however, antioxidants must possess the ability to neutralize all the various ROS entities that are directly involved in skin damage. These especially include hydroxyl (•OH) and peroxyl (ROO•) species as free radicals, hydrogen peroxide (H_2_O_2_) as a neutral molecule, singlet oxygen (^1^O_2_) as an electronically excited species and peroxynitrite (OONO^−^) as a reactive anion. While ROS also includes superoxide anion (O_2_^•−^), it is generally much less reactive than other ROS; however, it can reduce ferric and cupric ions that react with H_2_O_2_ via the Fenton reaction to give rise to •OH as a more reactive ROS [33].

While the skin’s intricate network of enzymatic and non-enzymatic antioxidant defenses has the ability to neutralize ROS, prior work has documented that even low exposures to environmental stressors generate such an abundance of ROS that innate defenses become easily overwhelmed. For example, even exposures to the sun that are insufficient to cause erythema are sufficient to induce an abundance of ROS within the skin. When this occurs, ROS are free to attack and damage the skin, creating oxidative stress within it [15].

**Figure 3:**
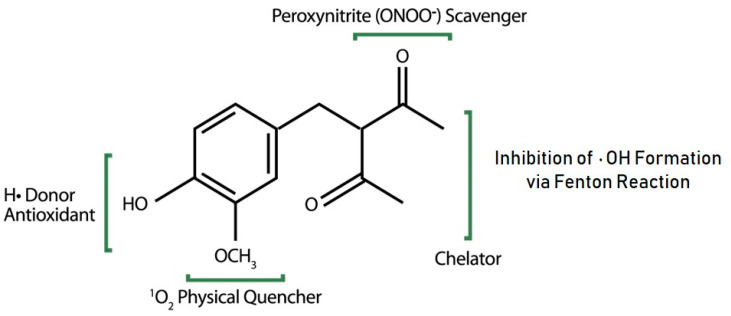
Roles played by different functional groups on AZ (adapted from Chaudhuri et al. [22]).

The key mode of action that antioxidants commonly conjure up relates to their ability to donate an electron or hydrogen atom to neutralize free radicals containing an unpaired electron, which is typically provided by a phenol moiety [34]. While this represents an important property, antioxidants also must be equipped with additional structural features to neutralize other reactive forms of ROS, especially singlet oxygen and peroxynitrite, two of the most potent oxidants formed within biological systems [35,36]. The addition of an acetyl group to afford a 1,3-dicarbonyl functional group on AZ creates a unique chemical structure that features a phenol group, an electron-rich aromatic ring and an enolizable di-carbonyl side chain. As reported previously [22], these functional groups together endow AZ with exceptional properties to neutralize all the most reactive forms of ROS, chelate iron and copper ions to prevent formation of •OH via the Fenton reaction and intercept excited state species of endogenous chromophores to prevent upstream ROS formation in sun-exposed skin (Figure 3).

### 4.1. Direct Neutralization of ROS

The antioxidant properties of AZ have been assessed using the same battery of chemical assays that is commonly utilized in the industry to measure the antioxidant effectiveness of single ingredients or mixtures of ingredients from botanical extracts [22]. These assays established that AZ was significantly more effective than α-tocopherol in neutralizing •OH, ROO•, OONO^−^ or ^1^O_2_ by factors of 17.7, 39.6, 126 or 2.7, respectively. Interestingly, in addition to outdoor environmental stressors, attention has been recently drawn to ozone as an aerial pollutant and source of •OH within indoor environments with added implications for the effects of oxidation in human health [37]. These results hold relevance for the skin since α-tocopherol is the chief constituent of vitamin E found in human skin and since vitamin E is the main lipophilic non-enzymatic antioxidant of the skin’s innate defenses. Moreover, depletion of vitamin E upon exposure of skin to environmental stressors has been shown to represent an early and sensitive in vivo biomarker of oxidative stress [38].

Two other salient findings from these model reactions also underscored the effectiveness of AZ as a multi-functional antioxidant [30]. First, the findings revealed that AZ deactivates ^1^O_2_ predominantly via physical quenching as opposed to chemical scavenging, in contrast to α-tocopherol which functions mainly through chemical scavenging. Physical quenchers restore ^1^O_2_ back to its original ground state (^3^O_2_) without being consumed in the process, whereas scavengers work through a chemical reaction and as a result become consumed and sacrificed. Many organic compounds typically deactivate ^1^O_2_ through a combination of both physical quenching and chemical scavenging; however, physical quenching appears more favorable for electron-rich aromatics containing phenol or methoxy groups, both of which reside on AZ [39,40]. The non-sacrificial nature of physical quenching bestows additional advantages to AZ since it can deactivate significantly more molecules of ^1^O_2_ than other conventional antioxidants that function via chemical scavenging. Secondly, AZ displays high efficiency to scavenge OONO^−^, which aligns with the presence of the 1,3-dicarbonyl group on the aromatic ring. Prior work documents that peroxynitrite shows high reactivity toward enolizable carbonyls, either through one-electron oxidation or nucleophilic addition reactions [41].

### 4.2. Selective Chelation of Fe^2+^ and Cu^2+^

The interaction of Fe and Cu ions with excess levels of H_2_O_2_ via the Fenton reaction is recognized as an important source of •OH within the skin [9]. This species of ROS is highly reactive and is a main initiator of lipid peroxidation. Normally, the levels of •OH are kept in check through the action of the skin’s major endogenous antioxidant defense enzymes, superoxide dismutase (SOD) and catalase (CAT). SOD dismutates O_2_^•−^ to H_2_O_2_ and then CAT reduces H_2_O_2_ to water [42]. In this way, the skin’s endogenous antioxidant enzymes help protect cells against •OH toxicity. However, excess levels of Fe^2+^ and H_2_O_2_ commonly occur in human skin because of exposure to environmental stressors such as UVA radiation. This exposure to UVA triggers immediate release of Fe^2+^ within cells from the iron-storage protein ferritin [43] and stimulates increased levels of H_2_O_2_ in stratum corneum and dermal fibroblasts by attenuating CAT but not SOD activities, which cause imbalances in the CAT/SOD ratios that lead to H_2_O_2_ accumulation [44,45].

Through the di-keto tautomer of the 1,3-dicarbonyl moiety, AZ has been shown to chelate Fe^2+^ with high efficiency. In simple model reactions between Fe^2+^ and H_2_O_2_, AZ was found to abolish production of •OH and was significantly more effective than EDTA as a common chelator and even more effective than Deferiprone, a pharmaceutical active used for chelation therapy and one of the most potent known iron chelators [46].

### 4.3. Physical Quenching of Key Excited State Chromophores in Skin

An abundant source of ROS within the skin originates from its exposure to solar radiation. Numerous studies confirm that both UV and visible spectral regions of solar radiation induce significant levels of ROS formation within the skin, with at least 50% of ROS originating from short-wavelength visible radiation [10,47]. Solar radiation exerts its effects in the skin through endogenous chromophores in cells or extracellular matrix, which absorb incident radiation and as a result form excited state species that subsequently sensitize production of ROS via Type I or Type II oxygen-dependent reaction pathways. In Type I pathways, excited state species interact directly with biomolecules (such as DNA) through electron donation or abstraction to form radicals that subsequently react with molecular oxygen to generate ROS, whereas in Type II reactions, the excited state species interact directly with molecular oxygen (O_2_) via energy transfer to form singlet oxygen (^1^O_2_) [48]. Many endogenous chromophores within the skin capable of absorbing UV and visible light have been identified along with the specific ROS that they have been found to sensitize, including melanin, NADPH oxidase, lipofuscin, porphyrins, riboflavin, and urocanic acid, among others [49].

With their clear link to excess ROS formation and oxidative stress, excited states of endogenous chromophores that form within the skin during exposure to solar radiation have emerged as molecular targets for upstream ROS prevention [50]. Advanced glycation endproducts (AGEs) are one of the main groups of heterogenous chromophores that comprise potent sensitizers of photooxidative stress in skin [51]. AGEs are derived from non-enzymatic reactions between amino groups on side chains of lysine and arginine residues in proteins and reactive carbonyl compounds, including reducing sugars such as glucose and other reactive carbonyl species such as malondialdehyde, glyoxal, methylglyoxal or glucasone that arise as byproducts of lipid peroxidation, glycolysis, or sugar oxidation. AGEs are formed intracellularly and extracellularly but are especially abundant on collagen and elastin in the dermis, where they accumulate with chronological age as well as in the skin regularly exposed to the sun over the course of life [52]. AGEs absorb UVA radiation forming excited states that sensitize the formation of O_2_^•^, H_2_O_2_ and ^1^O_2_ [51].

As discussed previously, excited state species can be intercepted by molecules acting as physical quenchers. In this mechanism, physical quenchers directly accept the energy of excitation from endogenous chromophores such as AGEs before they can form ROS, thereby restoring excited chromophores to their original ground states. At the same time, the excess energy acquired by physical quenchers elevates them into excited states, which they subsequently dissipate as heat. Once physical quenchers dissipate their excess energy, they can continue to relax more excited chromophores in the same fashion. In this way, physical quenchers are self-regenerating and are not sacrificed or consumed in the process in contrast to traditional antioxidants, which confers extra advantages in skin photoprotection from excess ROS generation [50,51].

Interestingly, the performance of physical quenchers can be distinguished from that of antioxidants with a specially designed assay based on suppression of cleavage of plasmid DNA containing glycated bovine serum albumin as a representative AGEs under irradiation with UVA [50,51]. In this assay, plasmid DNA is cleaved equally well in the presence or absence of molecular oxygen, indicating that the only way to inhibit cleavage is to intercept the excited states of AGEs before they can directly interact with plasmid DNA. When added to the assay prior to irradiation, AZ inhibited plasmid DNA cleavage by 85%, which confirms that AZ has the capability to interfere with AGE photosensitization. That AZ has been shown to deactivate ^1^O_2_ predominantly through physical interaction without chemical depletion also implies that AZ could inactivate AGE-excited states through physical rather than chemical quenching. In this regard, AZ performs similarly to L-proline and its ester derivatives, compounds that are also identified in this screening assay and recommended as novel physical quenchers for extra protection against photooxidative stress [50].

**Figure 4:**
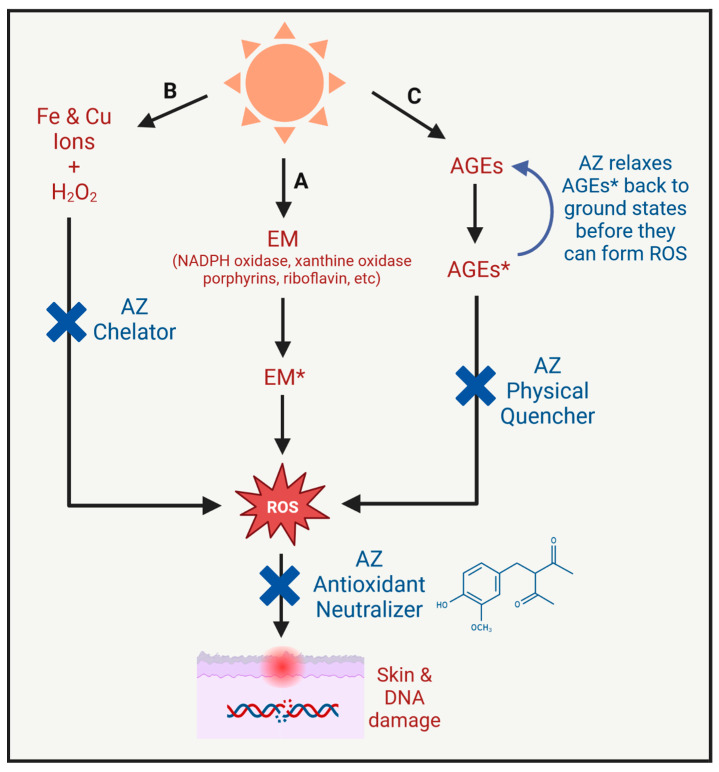
Summary of multiple pathways by which AZ manages overproduction of ROS in skin with sun as the key environmental stressor. A: AZ as a proficient antioxidant neutralizer that captures ROS formed from excited states of various endogenous molecules (EM*) before they elicit skin damage; B: AZ as a selective chelator of iron and copper ions to inhibit production of hydroxyl radicals through the Fenton reaction; and C: AZ as an effective physical quencher of advanced glycation endproducts (AGEs) as one of the most powerful sources of ROS formation contributing to photooxidative stress in skin.

In summary, AZ’s unique chemical structure equips it with the ability to manage excess ROS formation and contribute to the skin’s antioxidant defenses through multiple pathways (Figure 4), including (1) direct neutralization of the most reactive forms of ROS; (2) predominate deactivation of ^1^O_2_ through physical quenching; (3) inhibition of •OH formation through selective chelation of Fe^2+^; and (4) physical quenching of excited-state AGE, one of most potent mediators of photooxidative stress identified within the skin.

### 4.4. Management of Excess ROS Levels by AZ Translates into Biological Systems

The proficiency of AZ to manage excess ROS levels in biological systems has been demonstrated in keratinocytes by monitoring the extent to which AZ modulated intracellular build-up of ROS when cells were exposed to real-world doses of environmental stressors, including UVA, blue light, or urban dust as a form of particulate matter [22,46]. In all cases, exposure of cells in the absence of AZ to each environmental stressor significantly increased ROS levels by several factors relative to unexposed control cells. Pre-treatment of cells with AZ, however, prior to being stressed with blue light or particulate matter, reduced ROS levels in a dose-dependent manner, with even the lowest dose (31.2 µg/mL) providing significant reductions whilst higher doses (125 and 250 µg/mL) restored ROS levels to those of unstressed control cells [46]. Similarly, incubation with 25 or 50 µg/mL of AZ prior to UVA exposure reduced intracellular ROS levels by about 35% and 46%, respectively [22]. In agreement with these results, pre-incubation of cells with AZ over similar doses was also found to reduce lipid peroxidation in keratinocyte membranes by about 62–88% when the cells were stressed with particulate matter [46]. The fact that AZ does not absorb UV or visible radiation and therefore cannot function through a sunscreen-type effect further reinforces AZ’s unique properties to attenuate excess ROS formation in cells [22].

Overall, these results (1) certify that AZ continues to function as efficiently in biological systems as it does in chemical assays; (2) emphasize that AZ is highly efficient in protecting cells against the build-up of excess ROS induced by exposure to environmental stressors most implicated as causes of premature skin aging; and (3) highlight the ability of AZ to intercept ROS in biological systems once formed but before they can attack and inflict damage to major biomolecules such as lipids; and (4) confirm that AZ itself does not act as a photosensitizer in cells upon exposure to UV or blue light since otherwise it would increase rather than decrease the intracellular ROS levels. This latter point is important as it has been documented that many compounds, especially botanical extracts, while efficient in chemical assays, can become strong photosensitizers under UV or visible radiation [49].

## 5. AZ Abrogates Ongoing CPD Formation

One of the most salient observations in photobiology in recent years was that the formation of cyclobutane pyrimidine dimers (CPDs) continues to occur in the skin for several hours in the dark after UV exposure stops. CPDs represent the main class of DNA lesions that correlate strongly with induction of melanoma and non-melanoma skin cancers and arguably characterize the most important type of photodamage caused by sun exposure. Ongoing formation of CPDs in the dark (dCPDs) was first reported in melanocytes [53] and more recently in keratinocytes both in vitro and in vivo [54,55].

The striking feature of ongoing generation of dCPDs is that it occurs through a novel mechanism that invokes chemiexcitation. Chemiexcitation refers to the generation of excited state species at the expense of chemical reactions as opposed to direct absorption of UV or visible radiation that forms the basis of photochemistry. CPDs arise from excited state species of DNA (DNA*), which until recently were thought to originate in mammalian cells solely from the absorption of UV radiation. However, it is now appreciated that DNA* can arise through an alternative pathway in the absence of UV radiation via chemiexcitation. To date, this alternative chemiexcitation pathway to CPDs has been best characterized in melanocytes. Briefly, after UV exposure stops, sustained peroxynitrite (OONO^−^) oxidizes melanin to generate 1,2-dioxetanes as high-energy intermediates, which subsequently decompose to generate excited state triplet carbonyls as a product. With high energy, such excited state triplet carbonyls can transfer their energy to DNA to produce DNA*, the key entity that precedes the formation of CPDs. Interestingly, dCPD formation occurs to a significant extent not only after irradiation but also during irradiation in melanocytes. In fact, at any time point after UV-exposure, it is estimated that the chemiexcitation pathway accounts for about 50% of total CPDs created in melanocytes, which illustrates the significance of the chemiexcitation pathway and potentially suggests a new role for melanin in cancer development [53].

While they constitute effective defenses against erythema and while numerous studies document their ability to inhibit CPD formation (iCPDs) during sun exposure [56,57], sunscreens lack the ability to intervene in ongoing dCPD formation once sun exposure stops. This stems from the observation that sunscreens only exert protective benefits by functioning on the skin’s surface layers to absorb and neutralize harmful UV radiation before it can reach and damage the underlying skin. Moreover, sunscreens have no capacity to neutralize ROS or to prevent ROS from forming within skin upon exposure to UV radiation [49,58]. These shortcomings highlight the need for additional additives to bolster protection against dCPD formation not only in sunscreens but in all topically applied products.

Additional studies have been completed to investigate the ability of AZ to protect against dCPD formation. In one study, when added to pigmented melanocytes immediately after UVA exposure, AZ (25 µg/mL) inhibited ongoing dCPD formation by about 82% within the first hour post-UVA exposure, confirming the ability of AZ to intervene effectively in the chemiexcitation pathway of dCPD formation [22]. In this study, AZ’s effectiveness was attributed to its efficient properties to scavenge OONO−, chemically reduce 1,2-dioxetane intermediates or physically quench the excited state triplet carbonyls that form from the thermal decomposition of the 1,2-dioxetane intermediate, all of which would reduce the formation of DNA* and hence inhibit dCPD formation. That AZ was added to melanocytes post-UVA exposure, coupled with the fact that AZ does not absorb UVA radiation, also supports a mechanism in which AZ does not function through a sunscreen-type effect.

In a separate study, AZ and several of its chemical analogs [59] were evaluated to probe the AZ’s mechanism of action in pigmented melanocytes in greater detail. From structure–activity relationships, this study ruled out the possibility that AZ’s efficacy stems from reduction in 1,2-dioxetane intermediates but did establish that the integrity of the pentane-2,4-dione group on AZ was essential to its ability to block dCPD formation. In addition, the study found that AZ could reduce dCPD formation by upregulating nucleotide excision repair (NER) enzymes, which predominantly repair CPD lesions, or by inhibiting nitric oxide synthase (NOS) activity. Inhibition of NOS lowers the production of nitric oxide (NO•), which is a critical precursor along with superoxide anion (O_2_^•−^) in the formation of OONO^−^. Stimulating CPD repair or lowering OONO^−^ levels would also reduce dCPD formation. In conjunction with previous observations, the results support the hypothesis that AZ functions through multiple routes to block dCPD formation in pigmented melanoctyes, including (1) directly scavenging OONO^−^, (2) physically quenching intermediate formation of triplet carbonyls, (3) upregulating NER enzymes or (4) inhibiting NOS activity. Interestingly, physical quenching of triplet carbonyls by plant polyphenolic compounds has drawn considerable attention recently as a mechanism to prevent dCPD formation [60]. Thus, as was the case in chemical assays, these results highlight the capability of AZ to exert multifunctional properties in biological systems to reduce dCPD formation.

## 6. AZ Alters the Expression of Genes Contributing to Age-Related Extracellular Matrix (ECM) Dysregulation, Cellular Senescence and Inflammaging

Repeated daily exposure to exposomal factors (sun, pollution, climate, tobacco) and their complex interplay drive premature or extrinsic aging of skin throughout life. Visible signs of premature aging (coarse wrinkles, roughness, sagging, loss in elasticity, uneven pigmentation) overlay those of natural intrinsic aging (skin thinning, dryness, fine wrinkles) and make the skin appear older than its actual chronological age [1,2,3,4]. Of all environmental stressors, sun exposure may account for as much as 80% of the visible signs of facial aging (i.e., photoaging), while exposure to air pollution in the form of particulate matter correlates with hyperpigmentation and wrinkle formation [61,62]. The clinical signs of premature aging are closely associated with major alterations and breakdown of collagen and elastin, two of main constituents within the extracellular matrix (ECM) of the dermis. These include reductions in collagen levels, owing to self-perpetuating cycles of degradation and inhibition of de novo synthesis, plus accumulation of abnormal, non-functional elastotic material called solar elastosis. Together, these alterations in collagen and elastin lead to loss in skin stiffness, resiliency, and elasticity, which manifest clinically as sagging, wrinkles, and laxity in skin’s appearance, which have long been considered hallmarks of photoaging [63].

### 6.1. Alteration of Age-Related ECM Dysregulation

Numerous studies over the past years have correlated oxidative stress and excess ROS production with increased expression of matrix metalloproteinases (MMPs) that have capacity to degrade ECM proteins [64,65]. More recently, attention has been focused on the important role that ECM provides in regulating fibroblast function [65,66,67]. From this viewpoint, the chronic burden of ROS produced in the skin from both environmental stressors and intrinsic sources induce continual collagen fragmentation and disorganization, which decreases fibroblast attachments to the ECM scaffold, collapses their size and affects their function. These physical changes in fibroblast morphology stimulate further ROS and oxidative stress within the cells to promote increased production of multiple MMPs, increased levels of the matricellular protein CCN1 (cellular communication network factor 1) and decreased TGF-β (transforming growth factor β) signaling. This ultimately leads to the development of an age-associated dermal microenvironment (AADM) characterized by increased collagen breakdown, elevation of inflammatory cytokines (inflammaging) and inhibition of collagen synthesis. Recently, the collective term “matrisome” was used to refer to the set of genes contributing to dermal ECM composition, which can be subcategorized into collagens, proteoglycans, secreted factors, ECM glycoproteins, ECM regulators and other ECM-affiliated proteins [68,69]. This has provided a framework in which the foundations of human skin aging can be viewed as a disorder of matrisome components.

Gene expression analyses evaluating the effects of AZ conducted with microarrays in reconstituted human epidermis have suggested mechanisms by which AZ may improve or maintain ECM integrity [70]. AZ bolstered synthesis of mRNAs encoding genes from several core matrisome categories, including collagens (*COLL11A2*, *COL6A3*, *COL11A1*), proteoglycans (*OGN*, *VCAN*, *HSPG2*), and ECM glycoproteins (*SPARC*, *MGP, GLDN*, *NOV*). These effects of AZ on ECM components could be attributed to the unique chemical structure of AZ, since they were not observed in the tissue treated with zingerone, a molecule sharing some features with AZ but without a 2,4-pentadione branching chain. Among genes encoding matrix metalloproteinase enzymes, only the expressions of MMP3 and Cathepsin V (CTSV), a member of the peptidase C1 family, were down-regulated by AZ; however, in vitro enzyme assays demonstrated the inhibition of the MMP-1, MMP-3 and MMP-12 proteins. These studies indicate that AZ may maintain collagen structure via two key mechanisms, first by improving de novo collagen synthesis and second by preventing collagen degradation through MMP inhibition.

### 6.2. Alteration of Cellular Senescence

Replicative senescent dermal fibroblasts have been utilized as an in vitro aging model, with defects in collagen homeostasis that resemble those seen in aged human skin [71]. Comparisons between the AZ gene expression signature and those seen during fibroblast senescence have demonstrated a negative correlation, with AZ eliciting a decreased expression of senescence-induced genes (e.g., *SERPINB2*, *MMP3*, *LYPD6B*, *NPAS1*) and an increased expression of senescence-repressed genes (e.g., *OLFML3*, *TMEM119*, *LAMA4*). Many of the senescence-decreased genes up-regulated by AZ were associated with mitosis and microtubule organization, whereas senescence-increased genes down-regulated by AZ were frequently related to cell communication and signal transduction. AZ thus inhibited gene expression patterns associated with fibroblast replicative senescence, the long-term presence of which contributes to skin aging by promoting a senescence-associated secretory phenotype (SASP) that decreases proliferation and activates MMPs.

### 6.3. Alteration of Inflammaging

**Figure 5:**
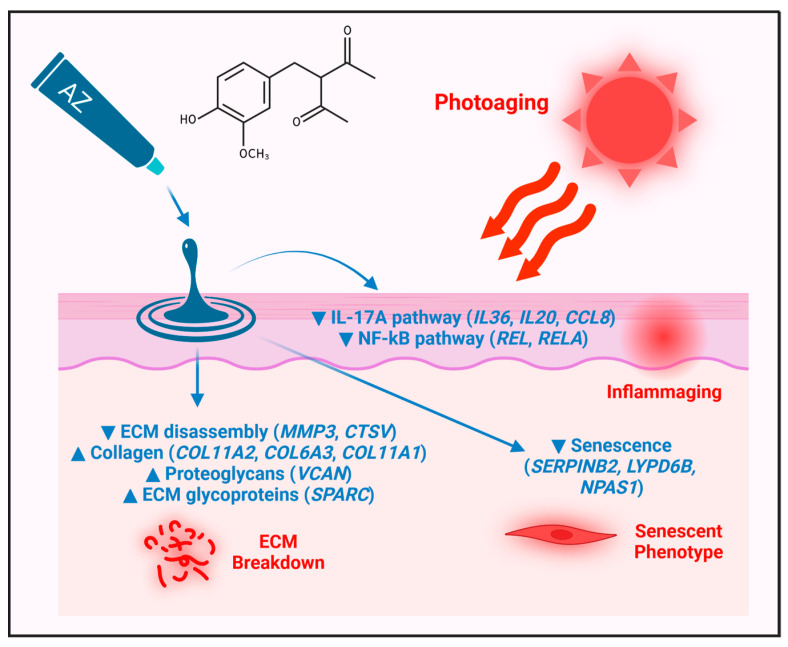
Photoaging and hypothesized mechanisms of protection by AZ. Long-term sun exposure leads to extrinsic skin aging and associated age-related inflammation (i.e., inflammaging), acceleration of fibroblast senescence and, ultimately, breakdown of ECM and the dermal matrix. The application of AZ is hypothesized to combat these effects by inhibiting the IL-17A and NF-kB pathways in the epidermis, down-regulating genes associated with fibroblast senescence, and by orchestrating a shift in the expression of matrisome-associated genes. Such matrisome-related genes are involved in diverse aspects of ECM function, including ECM disassembly, collagen production, and the activity of proteoglycans and ECM glycoproteins.

The accumulation of senescent dermal fibroblasts in aged skin not only contributes to ECM degradation but additionally promotes the release of proinflammatory factors that contribute to inflammaging [72,73]. Expression profiling has identified possible anti-inflammatory mechanisms of AZ, including decreased expression of genes contributing to inflammatory response and chemokine-mediated signaling (e.g., *CXCR4*, *CCL8*, *CCL3*, *BMP2*). Moreover, there was an overall negative correlation between effects of AZ on gene expression and those seen in KCs treated with IL-17A, a cytokine with central roles in inflammation and skin diseases such as psoriasis. Notably, AZ decreased expression of two cytokine-encoding mRNAs stimulated by IL-17A treatment, *IL36G* and *IL20*, suggesting that AZ may inhibit disease-associated cytokine networks triggered as part of the cellular IL-17A response. Moreover, AZ down-regulated genes encoding NF-κβ (*REL* and *RELA*) and the complete set of genes decreased by AZ overlapped with the genes up-regulated by *RELA* overexpression in HaCaT KCs. Overall, these results support the hypothesis that AZ exerts a nurturing influence to promote ECM homeostasis within the dermis, combined with an anti-inflammatory effect within the epidermis, suggesting mechanisms by which AZ can potentially combat skin aging or inflammatory disease (Figure 5).

## 7. AZ Promotes Stability of a Popular Ascorbic Acid Derivative Tetrahexyldecyl Ascorbate (THDC) Resulting in Improved Benefits to Skin

L-ascorbic acid (AA), also known as vitamin C, is essential for maintaining healthy and youthful-looking skin. AA promotes collagen production, improves barrier function and, as a constituent of skin’s innate antioxidant defenses, helps manage excess ROS production to attenuate oxidative stress [74]. Acquired through diet, deficiencies in AA intake are associated with many different types of skin conditions, including scurvy, hyperkeratosis, acne, and slow wound healing, among others [75]. Owing to its notorious chemical instability, however, the delivery of AA to the skin through topical preparations poses substantial formulation challenges [76]. To overcome these instability concerns, many skincare products use esters of AA, which act essentially as prodrugs that require conversion within the skin to release free AA for bioactivity [77]. Indeed, tetra-isopalmitoyl ascorbic acid (VC-IP), a lipophilic AA-ester was reported to undergo 84% conversion to free AA within reconstructed skin in 48 h [78].

One of the most popular AA-esters used today in cosmetic formulations is tetrahexyldecyl ascorbate (THDC), an ester that is structurally similar to VC-IP [79,80]. While THDC exhibits improved chemical stability compared with AA within formulation matrices and enhanced penetration through stratum corneum (due to its lipophilicity), it was reported recently that THDC is highly susceptible to facile degradation in the presence of ^1^O_2_, an entity of ROS that commonly forms within skin that is associated with significant levels of oxidative damage [81]. For example, exposure to ^1^O_2_ degraded TDHC by 94% within 2 min and completely within 6 min. These observations raise stability concerns around the efficacy of TDHC within oxidative microenvironments in the skin and suggest that TDHC may need to be accompanied by stabilizing antioxidants to prolong its lifetime.

With its proficiency in quenching of ^1^O_2_ predominantly through non-sacrificial physical interactions, AZ was found to help preserve the chemical integrity of THDC during ^1^O_2_ exposure. For example, in the presence of AZ, the existence of THDC was extended from 6.4% to 81% and from 0% to 75% after exposure to ^1^O_2_ for 2 or 10 min, respectively [81]. By extending THDC’s lifetime, it should be possible to increase the bioavailability of AA derived from THDC within skin to leverage its anti-aging and skin-brightening benefits. This idea was supported by observations from a gene expression profiling study, which showed that the effects of THDC differ in the absence or presence of AZ [81]. In the absence of AZ, unexpected pro-inflammatory effects of THDC were observed, including up-regulation of *IL1B* and genes associated with the type I interferon pathway (e.g., *MX1*, *MX2*, *STAT2*). These effects of THDC, however, were abrogated in the presence of AZ, and, in fact, the THDC+AZ combination decreased the expression of *IRF1* and the genes associated with chemokine response (e.g., *CCL2*, *ACKR4*, *CXCL6*). The THDC+AZ combination additionally decreased the expression of the genes encoding matrix metalloproteinase enzymes (e.g., *MMP1*, *MMP7*, *MMP14*) and replicated many of the gene expression changes seen during KC differentiation. Interestingly, the relative effects of THDC and its combination with AZ in cell-free assays showed that the combination of THDC+AZ led to a more potent inhibition of MMP-1, MMP-2 and MMP-3 enzyme activity compared to THDC alone [81]. These results support the idea that AZ and THDC have favorable synergistic effects, with at least part of the synergism derived from an increased stability of THDC when co-formulated with AZ.

While the above research strongly supports the need to co-formulate THDC with stabilizing antioxidants, it would be interesting to obtain additional confirmation on the benefits specifically for the combination of THDC with AZ in human skin in vitro by measuring THDC and AA levels within skin both before and after exposure to stressors known to increase the levels of ROS.

## 8. Translation of AZ’s Multiple Properties into Measurable Clinical Benefits

With the demonstration of its broad multifunctional properties in in vitro model studies ranging from proficient management of excess ROS to extra protection against delayed ongoing DNA damage to retinol-like ability to enhance collagen production within skin, the ability of AZ to provide measurable benefits to human skin in vivo has also been confirmed in key clinical studies.

### 8.1. AZ Improves the Appearance of Photoaged Skin

The application of topical products containing specialized ingredients, including plant-derived polyphenolic compounds, retinoids, or hydrating agents such as hyaluronic acid, can be an effective approach to mitigate the appearance of photoaged skin, which is commonly characterized by wrinkles, laxity, redness and dyspigmentation [82,83,84]. In this regard, AZ incorporated into a lotion at 1% was directly compared to its placebo lotion in a randomized double-blind clinical study where subjects applied either of the lotions twice daily to their face over a course of 8 weeks [85]. Compared to placebo, AZ lotion induced visible improvements to the appearance of facial photodamage, including significant reductions in wrinkle severity (−25.7%, *p* = 0.019), wrinkle volume (−30.1%, *p* = 0.003), dyspigmentation (−25.6%, *p* = 0.021) and redness intensity (−20.7%, *p* = 0.035). In addition, both lotions were well tolerated and neither caused any itching, burning or stinging sensations throughout the study.

### 8.2. AZ Supports Skin’s Microbiome in Composition and Diversity

Skin swabs taken from the nasolabial and glabellar skin regions of the face at the onset, and the conclusion of the photoaging study also revealed that the application of neither AZ nor placebo lotion negatively affected the composition or diversity of the skin’s microbiome over the study time course. In fact, AZ treatment (8 weeks) stimulated the skin-friendly *Staphylococcus epidermidis* by >20% vs. baseline, whereas placebo treatment remained practically unchanged. While this is a small study, these results spark interest for several reasons. First, recent research found that *S. epidermidis* plays an important role in maintaining the integrity of the skin’s barrier function by promoting the production of ceramides [86]. Second, *S. epidermidis* has been reported to be a main antagonist to *S. aureus*, a pathogenic organism frequently associated with cellulitis, wounds, and certain inflammatory skin conditions, including eczema, psoriasis and acne [87]. Third, because of their frequent application, the impact that personal care products exert on the diversity of the microbiome has emerged as an area of focus, with potential implications for the skin’s overall health and condition [88].

### 8.3. AZ Strengthens Defenses against UV Exposure

Intermittent or more acute sun exposures that unprotected skin receives during the normal course of daily routines or outdoor recreational activities throughout life have been identified as the single most important environmental stressor that drives premature aging of the skin [2,3,4]. The capacity of AZ to strengthen the skin’s defenses against the damaging effects of sun exposure was evaluated in a double-blind placebo-controlled clinical study, wherein a lotion containing 0.5% AZ was compared to placebo lotion. The primary outcome measure was erythema upon exposure to solar-simulated UV radiation (2 MEDs) after the products were applied to the skin twice daily over seven consecutive days (Chaudhuri, in preparation). Results showed that when two MEDs were administered using low or high irradiances to simulate chronic or acute exposures, the lotion containing AZ significantly reduced (*p* < 0.01) skin redness by 33% and 39%, respectively. That AZ itself exhibits minimal absorbance of solar-simulated UV radiation further emphasizes that AZ cannot provide these benefits through a sunscreen-type effect and must exert its protective benefits by strengthening the skin’s own natural defenses against the damaging effects of UV exposure.

## 9. Conclusions

Acetyl zingerone (AZ) is unique among skincare ingredients for its ability to deliver wide-ranging benefits to the skin. As a single photostable molecule, AZ can help the skin prevent and manage overproduction of ROS induced by continual exposures to environmental stressors, fortify defenses against ongoing epidermal DNA damage after sun exposure stops, and alter expression of genes that otherwise contribute to increased collagen breakdown, elevation of inflammatory cytokines and inhibition of collagen synthesis within the dermis. Moreover, through its proficient ability to neutralize singlet oxygen, AZ helps stabilize the ascorbic acid ester THDC and is thereby postulated to improve the bioavailability of AA within skin microenvironments. Additionally, THDC led to unexpected activation of type I interferon signaling, but this pro-inflammatory effect was suppressed by AZ. All these properties of AZ may translate to multidimensional skin benefits with topical application such as prevention of premature aging, protection against genotoxic insults leading to skin cancer, enhancing the skin’s own defenses against sun damage, and stimulating visual improvements to the appearance of photoaged skin. Thus, given that the skin is the first line of defense against the outer world and is inevitably exposed to diverse exogenous and endogenous stressors, the development of AZ as a specialized ingredient can provide a promising strategy to maintain skin’s health and appearance throughout the lifespan.

## Data Availability

Raw and processed microarray data have been submitted to the Gene Expression Omnibus (GEO) database (GSE133340 and GSE182177). All other raw data are available from the corresponding authors.
